# Psychogenic Non-Epileptic Seizures; a Narrative Review 

**Published:** 2020-01-20

**Authors:** Ameneh Jafari, Mostafa Rezaei Tavirani, Mohsen Parvareshi Hamrah, Sanaz Ahmadi Karvigh, Haniyeh Bashi Zadeh Fakhar

**Affiliations:** 1 Student Research Committee, Proteomics Research Center, School of Allied Medical Sciences, Shahid Beheshti University of Medical Sciences, Tehran, Iran.; 2 Proteomics Research Center, School of Allied Medical Sciences, Shahid Beheshti University of Medical Sciences, Tehran, Iran.; 3 Department of Neurology, Sina Hospital, Tehran University of Medical Sciences, Tehran, Iran.; 4 Department of laboratory science,chalous branch,islamic azad iniversity,chalous,iran.

**Keywords:** Seizures, Epilepsy, Psychogenic, Biomarkers, Conversion disorder

## Abstract

Psychogenic non-epileptic seizures (PNES) are paroxysmal changes that mimic epileptic seizures, so often misdiagnosed and treated for epilepsy. PNES are considered a psychiatric illness, personality pathology, and experiential and behavioral manifestation of depression. Despite studies over the past two decades, the pathological mechanisms of this disorder are unclear. In this paper, we critically review the current literature about the definition, epidemiology, diagnosis, treatment, related genes, and biomarkers of PNES and provide suggestions for future research. Further studies are needed for more information and knowledge on PNES to determine the appropriate psychotherapies and development of clear treatment guidelines.

## Introduction

Psychogenic non-epileptic seizures (PNES) are defined as paroxysmal changes in behavior, consciousness and autonomic function that resemble epileptic seizures (ES) but lack the electroencephalographic (EEG) signature of epileptic seizures [1-3]. PNES are characterized by disturbance of motor, autonomic, sensory, cognitive and/or emotional functions. The diagnosis of PNES is challenging and can take an average of 7 years between the manifestation of spells and definite diagnosis [4-6]. 

Therefore, patients with PNES are often misdiagnosed and face delays in reaching a definite diagnosis, and consequently are exposed to unnecessary antiepileptic drugs, emergency treatment, and even hospital admissions and other complications caused by unnecessary treatments [2, 7, 8].

PNES, erroneously diagnosed as epileptic also have an economic impact. The cost of misdiagnosis and treatment for PNES is surprising. Estimates suggest the cost of intractable epilepsy to be about $ 231,432 per patient in 1995 [4]. 

Considering the heterogeneity of patients with PNES, the etiology of this disorder is very diverse but some factors including trauma, neurological abnormalities, family dysfunction, stressful life events, and poor interpersonal skills and affect regulation, somatization, psychopathology, personality factors, and avoidant coping styles are identified as influential in development and maintenance of psychogenic non-epileptic seizures [3, 9, 10]. This review aimed to present an overview of existing research regarding the etiology, epidemiology, diagnosis, and management of PNES.

## Epidemiology

To understand the impact of PNES, various aspects other than absolute frequency should be considered. It must be noted that patients with PNES are as disabled because they are treated as a patient with epilepsy, receive high medical care and are consequently at risk of iatrogenic harm [11]. Epidemiological data on PNES are sparse and limited. One study from Iceland, estimated the incidence of PNES as 1.4 per 100.000, with symptoms commonly emerging in young adulthood (20 to 40 years old) [12]. However, it is important to keep in mind that PNES can be detected at any age; for example, they are not rare in children and are described in the elderly. Another study was demonstrated in Ohio and calculated the mean incidence of PNES at around 3/100,000 [12]. Approximately, 75% of patients with this condition are females, probably due to neurobiological, social and vulnerability differences [10]. Overall, although patients with PNES do not fit with a single stereotype, younger women with a high level of mental health and history of past abuse are more likely to be among this patient group. Prevalence of PNES is estimated in the range of one person per 30,000–50,000 or between 2-3.3 per 100,000 [13, 14]. 

Some studies have suggested that epilepsy may increase the risk of PNES not only through biological mechanisms but also by experiencing or observing epileptic seizure cases, which may provide an opportunity for model learning [15].

## Diagnosis

Early diagnosis of psychogenic non-epileptic seizures is very important, but unfortunately, the diagnosis is often delayed, and because of this, many patients are faced with the consequences of undesirable treatment [4, 12]. On the other hand, the diagnosis of PNES in the early phase may result in cost savings and reduce the burden on patients and the health care system [16]. Quality of life in PNES patients is significantly lower than in patients with epilepsy, which is due to psychopathology and undesirable effects of antiepileptic drugs [17].

Diagnosis of PNES poses particular challenges and PNES must be distinguished from other disorders, such as sleep disorders, migraine, hyperventilation, syncope, paroxysmal dyskinesia, movement disorders, transient ischemic attacks and myasthenia gravis, which are mediated by physiological or psychological causes [10]. An accurate clinical diagnosis of PNES is possible after history taking and direct observation of attacks by an epilepsy specialist in the majority of cases [10, 18]. The clinical and historical features that distinguish PNES from epileptic attacks are listed in table 1.

Some of the clinical features commonly found in people with epilepsy are often present in PNES. These include autonomic manifestations like tachycardia, incontinence, injury, flushing, sweating, nocturnal attacks and provocation of attacks by specific triggers such as flashing lights have often appeared to be a feature of epilepsy, but are commonly reported in PNES [19]. In addition to behavior during attacks, other characteristics of the patient's behavior can provide some clues and warn physicians of the possibility of PNES. For instance, they are more likely to have attacks in medical settings. PNES patients also seem to have a distinct way of communicating and talking about the attacks that distinguishes them from patients with epilepsy [20, 21].

Patients with PNES may be diagnosed with several psychiatric diseases including dissociative disorder, somatoform disorder, affective disorder, personality disorder, and other anxiety disorders [4].

Accurate diagnosis and management of PNES requires experience, skill, the use of video EEG and collaboration among neurologists, psychiatrists, pediatricians, nursing staff and other professional colleagues [1]. Approaches for diagnosis of PNES include epilepsy, EEG, symptom provocation, clinical history, and observation [22]. Video-EEG monitoring is considered a diagnostic gold standard in evaluation of seizure-like events, particularly in differentiating between PNES and epileptic seizures [7, 12]. Video-EEG is a highly specific and sensitive technique with a diagnostic yield of 50 to 60 percent. Although it is a costly technique, due to the intrinsic complexity of this group of patients, video-EEG confirmation is often needed for definitive diagnosis of PNES. In addition to Video-EEG, monitoring ictal eye closure and functional Magnetic Resonance Imaging (fMRI) might be useful methods for diagnosing patients with PNES [2]. Since EEG is an expensive method, it is not available in many centers, it also requires long-term admission and is not able to capture an actual seizure episode for several times, so alternative and auxiliary methods and indirect biomarkers are needed for diagnosis, which we will explain subsequently.

## Biomarkers

Multiple studies suggested various biomarkers for differentiation between epileptic seizures (ES) and PNES.

Evidence showed that Prolactin (PRL) values in ES patient groups were significantly higher than patients with PNES [23-25]. Results related to cortisol level are controversial. Zhang and Liu have demonstrated a typical pattern of pre-ictal decrease and postictal increase of cortisol in ES, but there were no such changes in PNES [26-28]. In their study, Bakvis et al. confirmed that PNES patients possess basal hyper-cortisolism, which positively correlated with both traumatic histories and threat vigilance [29, 30]. It is reported that a number of pituitary hormones such as thyrotropin-releasing hormone and growth hormone along with PRL and cortisol increase in serum following ES but not PNES [7, 31]. 

According to numerous studies, serum creatine kinase (CK) is the most promising candidate enzyme for differentiating ES from PNES with a sensitivity of 75%, and specificity of 85.5% [31-33]. Based on the evidence, raised serum creatine phosphokinase (CPK) level significantly correlates with the presence of a seizure as the cause of loss of awareness [34]. Javali and colleagues reported that none of the patients with PNES showed elevation in either serum PRL or CPK [34]. 

Concerning other hormone candidates, Ghrelin, Nesfatin and Brain-derived neurotrophic factor (BDNF) are important hormones that can be used as adjunct diagnostic tools for PNES [35, 36]. Aydin et al. have found postictal serum and salivary Nesfatin elevation in ES patients compared to those with PNES. Additionally, they detected lower postictal serum Ghrelin amounts in ES patients compared to those who had PNES [37]. Connolly and colleagues have demonstrated increased serum brain-derived neurotrophic factor (BDNF), a neurotrophin with important effects on neurogenesis and neuronal plasticity, in children with epilepsy; whereas lowered levels were detected in depressive disorder [38, 39].

Lafrance et al. confirmed lower postictal BDNF in patients with ES, similar to PNES patients; thereby, BNDF is not specific in differentiating seizure types. There is a hypothesis that decreased serum BDNF in ES and PNES is probably related to stress, not to seizure [40].

As we mentioned earlier, serious sexual, physical, or emotional abuse has been reported in childhood history of many PNES patients. Studies showed that PNES patients experience physical injuries to the hypothalamic–pituitary–adrenal (HPA) axis. Nearly 50% of people with PNES have psychological disorders related to trauma, including anxiety, depression and post-traumatic stress disorder (PTSD) [41]. Following activation of HPA axis, adrenocorticotropic hormone (ACTH) is secreted into the blood stream, which induces the release of cortisol. Ending the stress response is crucial because long-term activation of the sympathetic nervous system (SNS) and HPA axis can lead to enduring health consequences such as autoimmune diseases and growth disorders [42].

However, changes in other signaling pathways can also be attributed to stress-related health problems in PNES patients. For instance, higher levels of testosterone and lower levels of oxytocin have been reported in women with a history of abuse and chronic stress [43]. In women, estradiol is lower during stressful conditions compared to normal situations [44]. Moreover, increased amounts of PRL have been observed in post-ictal events in epilepsy [41].

Neuropeptide Y (NPY), another component of the SNS, is an inhibitory neuromodulator in the brain that can control propagation of limbic seizures. In addition, NPY's interaction with cortisol helps individuals cope with stress and maintain health, while overexpression and decrease of NPY are related to PTSD [41]. The role of NPY in PNES remains unclear. Decreased NPY levels may contribute to the onset of PNES symptoms. Studies have demonstrated lower levels of NPY and cortisol and elevated plasma ACTH levels in PNES patients with or without exposure to abuse in comparison to healthy controls [41, 45]. 

CNS, endothelial cells, and bone cells produce a member of the family of natriuretic peptide hormones called C-type natriuretic peptide (CNP) [46]. The concentration of CNP is very low in circulation and its half-life is also very short, about 3 minutes [47]. An amino-terminal fragment of CNP, NT-pro CNP, is more stable compared to its predecessor. Ceylan M et al. reported lower amounts of post-ictal serum NT-pro-CNP in ES patients compared to PNES patients and healthy controls, regardless of gender [48]. They postulated that such a difference is associated with CNP-related neural mechanisms, such as increased blood-brain barrier permeability, altered microcirculation, and synaptic stabilization [48].

## Associated factors

PNES occur in a heterogeneous population of patients. No single mechanism or interfering factor has been identified for PNES in all patients. Although past traumatic experience and physical or sexual abuses are a common feature and the main cause of PNES, numerous studies identified potential interacting factors in patients with PNES, including predisposing, precipitating and perpetuating factors. Predisposing factors such as sexual abuse and physical and emotional neglect will lead to individual vulnerability and the risk of developing PNES [15, 21, 29]. Precipitating factors can be divided into stressors or situations that would probably trigger attacks in patients with established PNES [15]. A wide variety of events, such as rape, death of or separation from family members or friends, job loss, injury, accidents, surgical procedures, natural disasters, tiredness, and relationship difficulties, seem to trigger the onset of PNES, because they occur days to months before the onset of seizures [22]. Factors such as anxiety, anger, depression, and mistreatment have been considered as potentially preventable perpetuating factors [15, 21]. In summary, the insight of the patient, as well as external factors that can enhance their behavior can be affected by confirming the PNES. All of these factors play an important role in the onset or development of PNES, and should not be ignored.

## Management and Treatment

Our knowledge remains limited to the best management strategies for PNES, and although several psychiatric interventions have been described, there is little evidence in this regard. This probably reflects the fact that PNES is not a disease entity but a symptom of a variety of basic psychiatric and psychological problems requiring a series of treatments [49].

In the initial phase of treatment (diagnosis delivery phase), effective communication with a neurologist is very important for a group of patients with PNES who often feel confused and irritated after diagnosis [50]. When the diagnosis of PNES is made, most patients are referred for psychological treatment. A wide variety of psychotherapeutic interventions including behavioral approaches, hypnosis, psycho-education, and family therapy have been proposed [4]. At the end of the second phase of treatment (engagement phase), patients will understand their diagnosis; no longer need other diagnostic evaluations elsewhere, start communicating with a mental health care provider, and actively participate in treatment [22].

The primary goal of the third phase of treatment (acute intervention phase) is to reduce the frequency of seizures, but improvement in psychiatric comorbidities, functional recovery, and quality of life is also desired [51].

Treatment will be selected according to the unique circumstances of the patient with PNES and will help resolve his/her emotional and psychological causes. Most patients with PNES have other psychiatric illnesses such as depression, anxiety, bipolar disorders, personality disorder, and many others. Of all the various interventions, Cognitive Behavior Therapy (CBT) significantly improved the range of clinical and psychological factors and was more effective in reducing attack frequency compared to standard medical care such as medication [52, 53]. Psychological training, relaxation education, exposure to inevitable conditions and cognitive restructuring are common techniques in CBT [52, 53].

The final phase of the treatment (long-term interventions phase) is particularly relevant to the subgroup of PNES patients who will need ongoing care to achieve functional recovery [22, 51].

**Figure 1:**
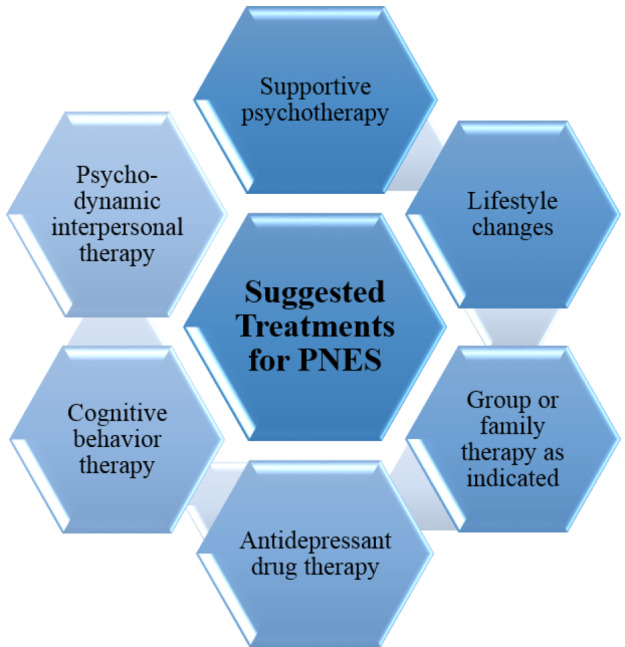
The main approaches for treatment of psychogenic non-epileptic seizures (PNES).

Some suggested treatments for patients with psychogenic non-epileptic seizures are illustrated in Figure 1. 

**Table 1:** Clinical and historical features suggested for diagnosis of psychogenic non-epileptic seizures (PNES)[10, 18]

**Clinical Features**	**Historical Features**
Observer’s ability to modify the patient’s motor activity	Associated (often multiple) psychiatric disorders
Avoidance behavior during seizures	Flurries of seizures or recurrent pseudo–status epilepticus that lead to multiple emergency department visits or hospitalizations
Asynchronous limb movements	
Change in symptomatology	High seizure frequency
Closed eyes during seizures or resisted eyelid opening	History of sexual or physical abuse
Dystonic posturing (including opisthotonos)	Lack of concern or an excessive or exaggerated emotional response
Emotional or situational trigger for seizure onset	Multiple unexplained physical symptoms
Ictal crying, weeping	No history of injury from seizures
If tongue biting is present, usually the tip (not the side) of the tongue	No response to antiepileptic drugs or a paradoxical increase in seizures with antiepileptic drug treatment
Intermittent or waxing and waning motor activity	
Gradual onset of attacks	Personal, family, or professional experience with epilepsy
Non-physiological progression	Seizures that occur only in the presence of others or only when the patient is alone
Prolonged seizure activity, duration of 2 or 3 minutes	
Rhythmic pelvic movements	
Out of phase movements	
Side-to-side head movements	

## Conclusion:

PNES include paroxysmal changes in responsiveness, behaviors, consciousness or movements that look like epileptic seizures but lack electrophysiological epileptic changes. PNES are common among women and usually begin in young adolescence. Diagnosis and treatment of these disorders are challenging. Accurate diagnosis and management of PNES need video-EEG monitoring and a multidisciplinary collaborative approach among pediatricians, psychiatrists, neurologists, and other specialists. In addition to a video-EEG, biomarkers have indicated limited benefit in diagnosis of PNES. Hyper-cortisolism, delayed CK/CPK release, lowered postictal serum and salivary Nesfatin and elevated postictal serum Ghrelin level are useful for differentiating PNES from ES. Furthermore, lower serum levels of BNDF and NPY are considered biomarkers for development of PNES. These biomarkers are useful for detection of PNES among patients with seizure symptoms, and easier and less expensive than video-EEG. 

Further studies are necessary to identify new biomarkers and psychopathological mechanisms for finding appropriate treatments for PNES in the future.
